# Assessment of fenofibrate-methylation interactions on triglycerides using longitudinal family data

**DOI:** 10.1186/s12919-018-0132-y

**Published:** 2018-09-17

**Authors:** Jih-Chang Yu, Fang-Chi Hsu, Yen-Feng Chiu

**Affiliations:** 1 Institute of Population Health Sciences, National Health Research Institutes, 35 Keyan Road, Zhunan, Miaoli, 35053 Taiwan; 2 Department of Biostatistical Sciences, Division of Public Health Sciences, Wake Forest School of Medicine, Medical City Boulevard, Winston-Salem, NC 27157 USA

## Abstract

**Background:**

Triglyceride (TG) concentrations decrease in response to fenofibrate treatment, and also are associated with DNA methylation. But how interactions between fenofibrate response and DNA methylation affect TGs remains unclear.

**Methods:**

In the present study, we identified and compared differential methylation sites associated with TG concentrations in individuals before and after fenofibrate treatment. We then estimated interactions between fenofibrate treatment and methylation to identify differential methylation effects associated with fenofibrate treatment on TG concentrations using the entire longitudinal family sample. To account for within-family and within-individual corrections, the generalized estimating equations approach was used to estimate main and interaction effects between methylation sites and fenofibrate treatment, adjusting for potential confounders. Analyses were also performed with and without adjusting for high-density lipoprotein (HDL) concentrations.

**Results:**

Prior to fenofibrate treatment, 23 cytosine-phosphate-guanine (CpG) sites were significantly associated with TG concentrations, while only 13 CpG sites were identified posttreatment, adjusting for HDL. Without adjusting for HDL, pretreatment, 20 CpG sites were significantly associated with TG concentrations, while only 12 CpG sites were identified posttreatment. Among these sites, only one differential site (cg19003390 in the *CPT1A* gene) overlapped from pre- and posttreatment measurements regardless of HDL adjustment. Furthermore, 11 methylation sites showed substantial interaction effects (*p* < 1.43 × 10^−7^with Bonferroni correction) with or without HDL adjustment when using the whole longitudinal data.

**Conclusions:**

Our analyses suggest that DNA methylation likely modified the effect of fenofibrate on TG concentrations. Differential fenofibrate-associated methylation sites on TGs differed with and without adjusting for HDL concentrations, suggesting that these HDLs and TGs might share some common epigenetic processes.

## Background

Blood lipid levels, including triglycerides (TGs) and high-density lipoproteins (HDLs) are heritable and modifiable risk factors for cardiovascular rand metabolic disease [1]. Although numerous genetic variants and genes have been associated with TGs and HDLs, these loci explain only a modest fraction of the observed variance [2]. DNA methylation is an epigenetic process involving the methylation of cytosine, usually at cytosine-phosphate-guanine dinucleotides (CpGs) in the promoter region or within genes. It plays an important role in gene regulation through influencing chromatin structures and changing coding regions for transcription [3]. Aging, diet, and exposure to metals all affect DNA methylation. Exposure to a number of chemicals also induces modification of cytosine, leading to its methylation [3]. Depending on whether a reduction or an addition in DNA methylation occurs, the sequence can either be hyper- or hypomethylated. Studying epigenetic contributions to TGs can help with the identification of relevant TG pathways and genes and, further, facilitate the design of new treatments and biomarkers for cardiovascular and metabolic diseases. Associations between levels of DNA methylation and TGs have been identified in epigenome-wide studies [4]. Methylation of CpGs has also been correlated with TG drugs [2]. However, how these associations are interrelated warrants further investigation. The present study aimed to assess the interactions between fenofibrate use and CpG methylation on TG concentrations using a pre- and posttreatment longitudinal study design. Differential sites with significant interactions are regarded as drug-associated methylations on TGs.

## Methods and materials

### Epigenome-wide association study and phenotype data

Association mapping was conducted using epigenome-wide association study real data from the GAW20 data set. A total of 463,995 whole-genome CpG methylation sites were assayed for 1105 individuals from 188 multiplex pedigrees [2]. A total of 114,240 CpG sites were not mapped to specific genes, resulting in 349,755 sites being included in the analysis. There were four TG and HDL measurements for each person. The first two measurements were obtained before fenofibrate treatment, while the last two were obtained posttreatment [5]. A total of 995 individuals from 182 pedigrees with pretreatment measurements and 530 individuals from 153 pedigrees with posttreatment measurements were included in the cross sectional analyses. A total of 421 individuals from 138 families with both pre- and posttreatment data were included in the longitudinal data analysis.

To assess fenofibrate and methylation effects on TGs, we used an average of the first two TG measurements as a pretreatment phenotype, and an average of the last two TG measurements as a posttreatment phenotype. As a result, all individuals had either or both pre- and posttreatment measurements for methylation and phenotypes. TG levels were log-transformed to approximate normality before averaging the first two or the last two measurements.

### Longitudinal data analysis

To identify differential CpGs interacting with treatment effects to influence TGs, we modeled TGs as a function of percent methylation, fenofibrate treatment, and their interactions at individual CpGs using the generalized estimating equation (GEE) approach [6]. Population stratification assessed by principal component analysis was minimal in this study population [5]. Therefore, we did not adjust for principal components in this study. We adjusted for covariates including age, sex, study site, and smoking at baseline in all analyses. Analyses were also run with and without adjusting for HDL concentrations. HDL concentrations were estimated identically to TG concentrations, by averaging the first two log-transformed HDL measurements and separately, the last two log-transformed HDL measurements. A dummy variable was created to indicate pre- and posttreatment (ie “without” treatment and “with” treatment, respectively). The following marginal model was used to analyze family data with repeated measures for the *m*^*th*^ methylation site:

*E*(*Y*_*ijr*_) = *α* + *β*^*T*^*X*_*ijr*_ + *εT*_*ijr*_ + *γG*_*ijrm*_ + *ηG*_*ijrm*_*T*_*ijr*_ where *Y*_*ijr*_ is the log-transformed TG; *X*_*ijr*_ is the vector of covariates as stated above; *T*_*ijr*_ is the dummy variable for treatment; *G*_*ijrm*_ is the percent methylation at the *m*^th^ methylation site for individual from family *i* at the *r*^*th*^ time point, *i* = 1, …, *N*,  *j* = 1, …, *n*_*i*_*, r = 1,2, m = 1,…,M; N* is the total number of families; *M* is the total number of methylation sites; and *α*, *β*^*T*^, *ε*, *γ* and *η* are the regression coefficients for the intercept, covariates, treatment, methylation, and drug–methylation interaction, respectively. We were interested in the significance of $\widehat{\eta}$, the estimate of interaction between methylation and treatment. Note that the dummy variable *T*_*ijr*_ and its interaction with methylation were not included in the model when conducting pre- and posttreatment analyses separately. The within-family and within-individual correlations were accounted for in the GEE approach [6]. An exchangeable working correlation structure was used in the analyses. All analyses were conducted using the statistical computing software package R 3.2.2.

To avoid over adjustment, correlations between HDL and TG were calculated. Correlation coefficients between the two log-transformed traits TG and HDL at pre- and posttreatment were − 0.448 and − 0.449, respectively. Thus, the overall average correlation coefficient was − 0.45.A Bonferroni-corrected genome-wide level 1.43×10^−7^ (0.05/349755CpGs) was used for the statistical significance threshold for the drug–methylation interaction.

## Results

**Fig. 1:**
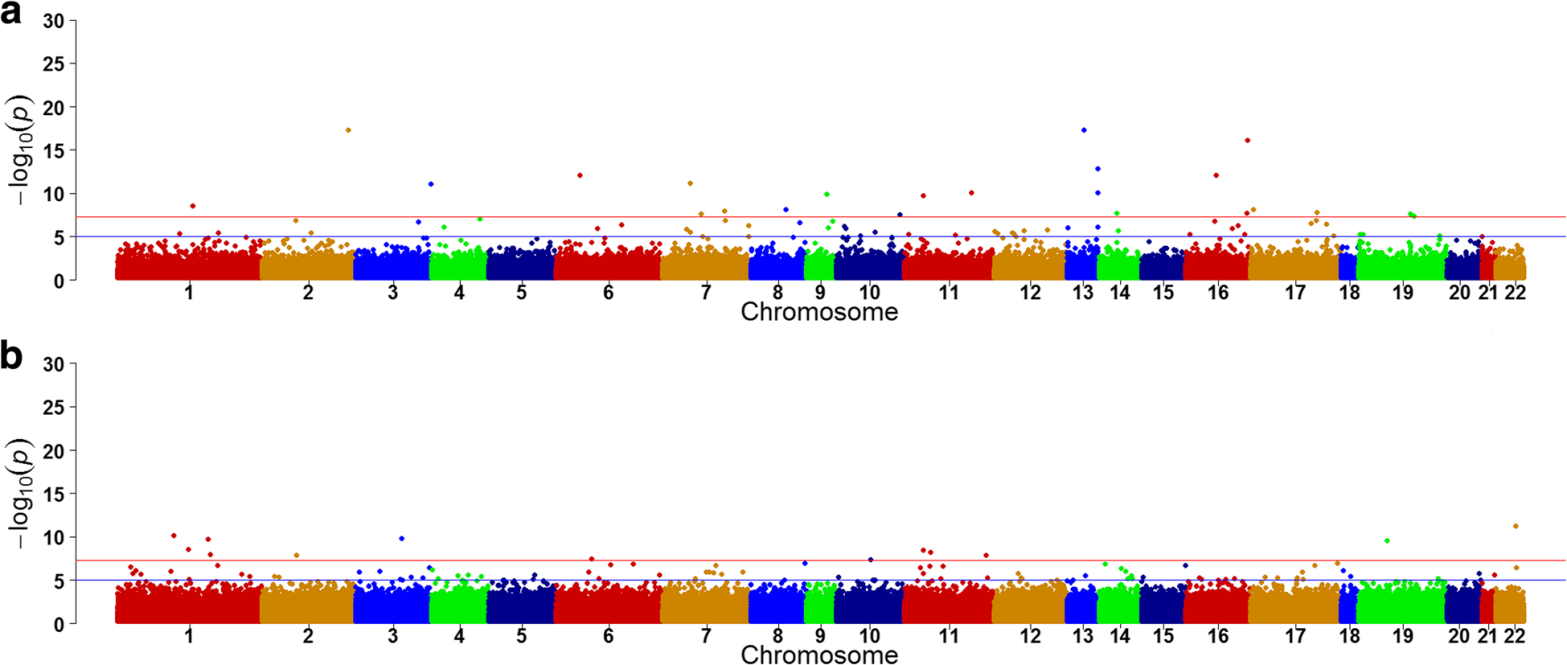
Differential CpGs associated with TG concentrations, after adjusting for HDLpre- (**a**) and posttreatment (**b**)

**Fig. 2:**
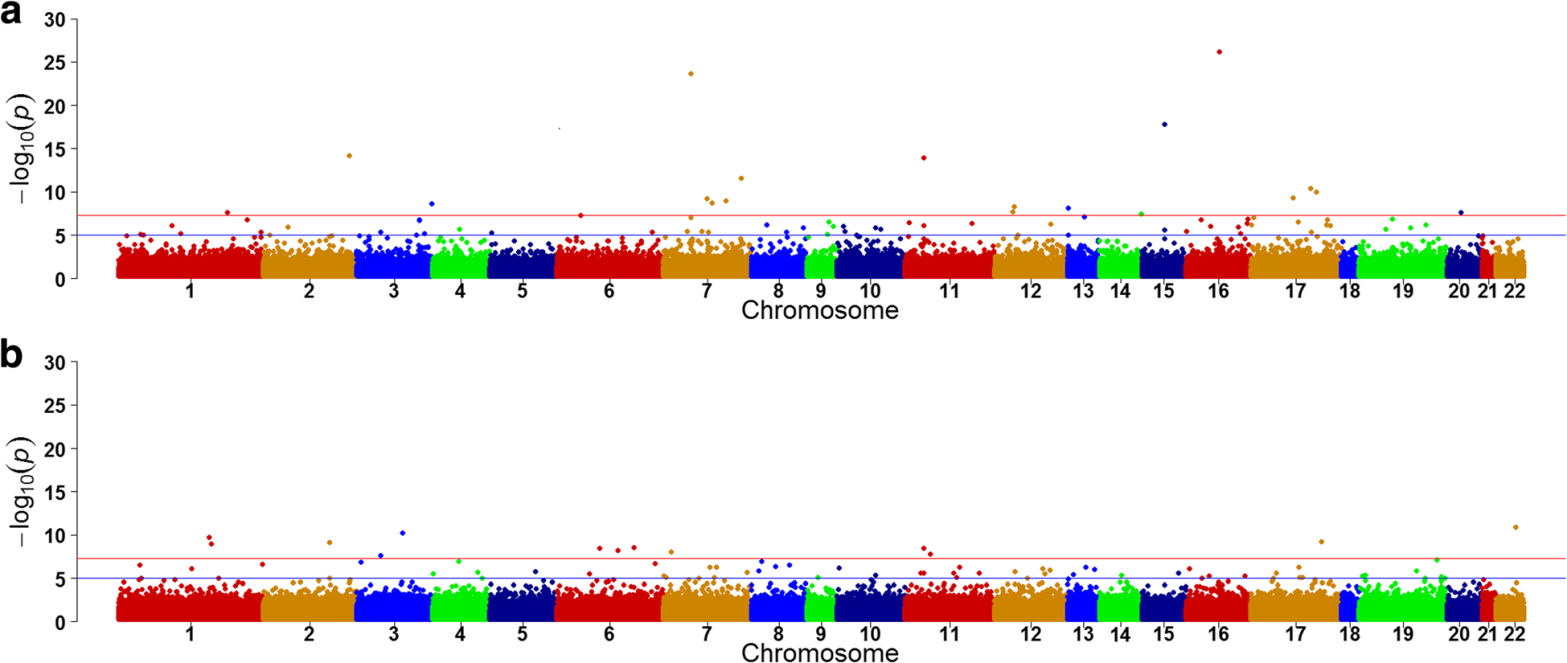
Differential CpGs associated with TG concentrations, without adjusting for HDL pre- (**a**) and posttreatment (**b**)

Figure 1 and Fig. 2 show differential CpGs associated with TG levels with and without adjusting for HDL from pre- (a) and posttreatment (b), respectively. With adjustment for HDL, before treatment, 23 CpGs were identified, whereas only 13 CpGs were identified posttreatment (Fig. 1). Without adjusting for HDL, before treatment, 20 CpGs were identified, whereas only 12 CpGs were identified posttreatment (Fig. 2). Among these sites, only one differential site (cg19003390 in the *CPT1A* gene) overlapped from pre- and posttreatment measurements, with and without adjusting for HDL. Among pretreatment measurements, 6 CpG sites were identified, with and without adjusting for HDL. On posttreatment, 5 sites were identified, with and without adjusting for HDL. These findings suggest that fenofibrate might alter TG concentrations, at least in part, through altering DNA methylation as well as through HDL.

**Fig. 3:**
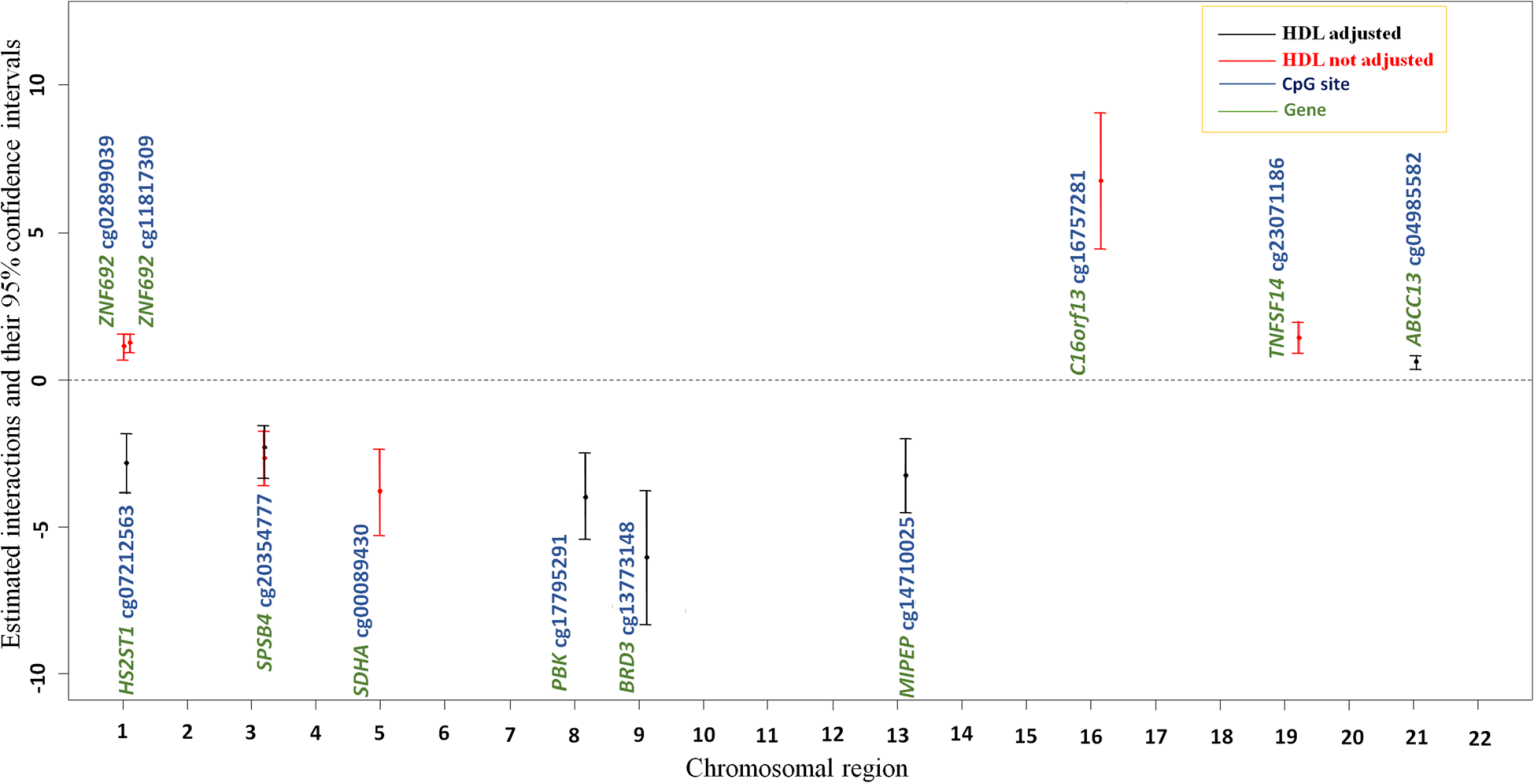
Comparisons for differential CpG sites with significant estimated regression coefficients for fenofibrate-methylation interactions, with and without adjustments for HDL concentrations

We further assessed interactions between treatment and individual CpGs with and without additionally adjusting for HDL (Table 1; Fig. 3) using the whole longitudinal sample. CpG sites with significant drug-associated impact on TG concentrations differed with and without adjusting for HDL, except cg20354777 of the *SPSB4* gene (Fig. 3). The differential sites with HDL adjustment included cg02899039, cg11817309, cg20354777, cg00089430, cg16757281, and cg23071186 on chromosomes 1, 1, 3, 5, 16, and 19, respectively (Table 1), suggesting that HDL might be a confounder for these interactions. In contrast, the differential sites without HDL adjustment included cg07212563, cg17795291, cg13773148, cg14710025, and cg04985582 on chromosomes 1, 8, 9, 13, and 21, respectively (Table 1). Only the cg20354777 site from the *SPSB4* gene was associated with TG concentrations regardless of HDL adjustment. The main effect of treatment, when excluding DNA methylation from the model, was substantially significant with an estimated effect of − 0.35 and a *p* value of 2.45 ×10^−53^ after adjusting for age, sex, study site, smoking, and HDL.

**Table 1:** Estimates of regression coefficients for methylation, fenofibrate, and methylation-fenofibrate interactions, with and without adjusting for HDL using the whole sample^a^

			With adjustment for HDL			Without adjustment for HDL		
Chr	Gene	CpG	Methylation (*p*)	Treatment (*p*)	Interaction (*p*)	Methylation (*p*)	Treatment (*p*)	Interaction (*p*)
1	*ZNF692*	cg02899039	**−0.16 (0.21)**	**−0.48 (1.11 × 10** ^**−60**^ **)**	**1.13 (8.14 × 10** ^**−8**^ **)**	0.24 (9.22 × 10^− 2^)	−0.35 (1.25 × 10^− 38^)	0.35 (2.27 × 10^− 2^)
1	*HS2ST1*	cg07212563	0.56 (0.13)	0.83 (0.13)	−1.36 (4.34 × 10^−2^)	**0.72 (4.24** × **10**^**− 2**^**)**	**2.28 (2.80×10** ^**− 7**^ **)**	**−2.84 (3.91 × 10** ^**− 9**^ **)**
1	*ZNF692*	cg11817309	**− 0.02 (0.45)**	**− 0.44 (1.90 × 10** ^**−71**^ **)**	**1.24 (1.24 × 10** ^**− 14**^ **)**	0.51 (2.03 × 10^− 5^)	−0.34 (1.38 × 10^− 47^)	0.35 (2.00 × 10^− 2^)
3	*SPSB4*	cg20354777	**0.48 (0.09)**	**1.77 (7.07 × 10** ^**−7**^ **)**	**− 2.68 (1.58 × 10** ^**− 9**^ **)**	**0.62 (1.94** × **10**^**− 2**^**)**	**1.56 (6.09 × 10** ^**− 7**^ **)**	**− 2.32 (2.38 × 10** ^**− 9**^ **)**
5	*SDHA*	cg00089430	**1.49 (0.02)**	**2.92 (3.42 × 10** ^**− 6**^ **)**	**− 3.81 (1.22 × 10** ^**− 7**^ **)**	1.07(3.52 × 10^− 2^)	2.20 (4.26 × 10^− 4^)	− 2.89 (6.20 × 10^− 5^)
8	*PBK*	cg17795291	0.28 (0.17)	0.34 (0.34)	−0.85 (0.17)	**0.45 (3.63** × **10**^**− 2**^**)**	**3.25 (5.91 × 10** ^**− 7**^ **)**	**−3.99 (3.70 × 10** ^**− 8**^ **)**
9	*BRD3*	cg13773148	1.42 (6.49 × 10^−3^)	3.79 (9.84 × 10^− 4^)	−4.60 (2.88 × 10^− 4^)	**1.40 (4.00** × **10**^**− 3**^**)**	**5.19 (4.00 × 10** ^**− 7**^ **)**	**− 6.04 (7.05 × 10** ^**− 8**^ **)**
13	*MIPEP*	cg14710025	−1.64 × 10^− 2^ (0.48)	0.89 (0.14)	− 1.48 (5.91 × 10^− 2^)	**0.36 (12.74** × **10**^**− 2**^**)**	**2.54 (2.65 × 10** ^**− 6**^ **)**	**− 3.26 (1.28 × 10** ^**− 7**^ **)**
16	*C16orf13*	cg16757281	**−3.24 (5.66 × 10** ^**−5**^ **)**	**−0.79 (7.07 × 10** ^**− 26**^ **)**	**6.75 (4.96 × 10** ^**− 9**^ **)**	− 2.90 (7.98 × 10^− 5^)	−0.63 (2.59 × 10^− 14^)	5.20 (1.11 × 10^− 5^)
19	*TNFSF14*	cg23071186	**−0.93 (7.90 × 10** ^**−5**^ **)**	**− 0.76 (3.87 × 10** ^**− 27**^ **)**	**1.42 (5.53 × 10** ^**− 8**^ **)**	−0.79 (2.18 × 10^− 4^)	−0.600 (1.75 × 10^− 22^)	1.09 (1.12 × 10^− 6^)
21	*ABCC13*	cg04985582	−0.35 (3.28 × 10^−3^)	− 0.75 (< 10^− 20^)	0.57 (2.80 × 10^− 6^)	**− 0.31 (2.20** × **10**^**− 3**^**)**	**−0.69 (< 10** ^**− 20**^ **)**	**0.61 (3.90 × 10** ^**− 9**^ **)**

^a^Table shows only CpGs with a *p* value for the interaction ≤1.43 × 10^− 7^ on log-transformed TG, adjusted for age, sex, study site, and smoking. The numbers in boldface refers to estimates and *p* values for the main and interaction effects where significant methylation-fenofibrate interactions were observed. A total of 421 individuals from 138 families with both pre- and posttreatment data were included in these analyses

## Discussion and conclusions

Differences in the identified CpG sites between pre- and posttreatment suggested that fenofibrate might alter TG concentrations, partially through altering DNA methylation. The CpGs identified pre- and posttreatment differed markedly. These findings indicate the existence of moderation effects from DNA methylation (or drug–methylation interactions) on TG concentrations. While DNA methylation does changes over time in individuals [7], in this study, time and drug effects confound each other and could not be distinguished in analyses. Only one methylation site, which was within *CPT1A*, showed an association with TGs pre- and posttreatment; other methylation sites were associated with TGs either pre- or posttreatment only. This finding confirmed the previous result that *CPT1A* methylation was strongly and robustly associated with TGs [2]. Furthermore, the interaction between the *SPSB4* gene and fenofibrate was significant regardless of HDL adjustment, suggesting that this interaction was independent of HDL. For *ZNF692, SDHA, C16orf13,* and *TNFSF14*, their drug–methylation interaction effects on TGs were significant only when adjusting for HDL, suggesting that HDL might be a confounder for these interactions. However, the drug–methylation interactions from *HS2ST1, PBK, BRD3, MIPEP*, and *ABCC13* were significant only when not adjusting for HDL. The interaction effects on TGs differed by the presence or absence of HDL adjustment, implying that TG and HDL are likely to share some epigenetic processes. Some genes with differential drug-associated CpG sites (drug–methylation interactions) on TGs, including *HS2ST1*, *ABCC13*, *ZNF692*, *SPSB4*, *SDHA*, and *TNFSF14*, were linked to metabolic risk factors or diseases (such as TG, glucose, HDL, type 2 diabetes, and ischemic heart disease) in previous studies [8–12]. *PBK* and *BRD3* were related to cancers and *MIPEP* was linked to left ventricular noncompaction, hypotonia, and infant death [13–15]. The correlations and interactions between fenofibrate treatment and CpGs on TGs reported here warrant further investigation.
